# Microcirculation abnormalities in patients with fibromyalgia – measured by capillary microscopy and laser fluxmetry

**DOI:** 10.1186/ar1459

**Published:** 2004-12-10

**Authors:** Susanne Morf, Beatrice Amann-Vesti, Adrian Forster, Ulrich K Franzeck, Renate Koppensteiner, Daniel Uebelhart, Haiko Sprott

**Affiliations:** 1 Department of Rheumatology, Institute of Physical Medicine, University Hospital, Zurich, Switzerland; 2 Department of Medicine, Division of Vascular Medicine (Angiology), University Hospital, Zurich, Switzerland; 3 Center for Vascular Diseases, Zurich, Switzerland

**Keywords:** capillary microscopy, fibromyalgia, laser fluxmetry, microcirculation

## Abstract

This unblinded preliminary case-control study was done to demonstrate functional and structural changes in the microcirculation of patients with primary fibromyalgia (FM). We studied 10 women (54.0 ± 3.7 years of age) with FM diagnosed in accordance with the classification criteria of the American College of Rheumatology, and controls in three groups (n = 10 in each group) – age-matched women who were healthy or who had rheumatoid arthritis or systemic scleroderma (SSc). All 40 subjects were tested within a 5-week period by the same investigators, using two noninvasive methods, laser fluxmetry and capillary microscopy. The FM patients were compared with the healthy controls (negative controls) and with rheumatoid arthritis patients and SSc patients (positive controls). FM patients had fewer capillaries in the nail fold (P < 0.001) and significantly more capillary dilatations (P < 0.05) and irregular formations (P < 0.01) than the healthy controls. Interestingly, the peripheral blood flow in FM patients was much less (P < 0.001) than in healthy controls but did not differ from that of SSc patients (P = 0.73). The data suggest that functional disturbances of microcirculation are present in FM patients and that morphological abnormalities may also influence their microcirculation.

## Introduction

Vasospastic symptoms occur in about 30% of patients with primary fibromyalgia (FM) [1]. These patients present with Raynaud's phenomenon and intolerance to cold [2]. Frodin and colleagues, using nailfold capillaroscopy in FM patients, found slight morphological changes, such as moderate enlargement of capillary loops and variations in calibre [3]. Jeschonneck and colleagues showed decreased microcirculatory blood flow above tender points in FM patients [4]. About 60% to 90% of systemic scleroderma (SSc) patients have Raynaud's phenomenon [5]. In patients with SSc other workers, using videomicroscopy with sodium fluorescein, have found typical changes of the nailfold capillaries, characterised by reduced capillary density, giant capillaries, avascular fields, microhaemorrhages, and disturbance of diffusion [6]. Furthermore, in rheumatoid arthritis (RA), peripheral malperfusion and vasculitis occur [7], resulting in skin ulcers, neuropathy, necrosis, or gangrene.

Our aim in this preliminary study was to investigate capillary abnormalities and blood flow by two independent objective methods, capillary microscopy and laser Doppler fluxmetry, to obtain evidence of disturbed microcirculation in FM patients.

## Materials and methods

The study group consisted of 10 women (54.0 ± 3.7 years of age) from the Outpatient Department in the Department of Rheumatology, University Hospital, Zurich, with primary FM classified in accordance with the criteria of the American College of Rheumatology [8]. The controls were three groups (*n *= 10 in each group) of age-matched women who were healthy (negative controls) or who had symptomatic RA or SSc (positive controls). The subjects were studied using laser Doppler fluxmetry and capillary microscopy.

None of the subjects had vasculitis. The RA patients did not have Raynaud's phenomenon and only 2 of the 10 had hypertension as a possible risk factor for small-vessel disease. Although the SSc patients did not have vasculitis, all except one presented with a typical Raynaud's syndrome, with a mean Medsger score [9] of 2.2 ± 1.03. This score describes how advanced the disease is (Table 1); a low scores (0 or 1) indicates no or mild SSc, and a high score (4) indicates the end stage. Smokers and patients treated with nitrate or Ca^2+^-channel blockers were excluded from the study. The study was approved by the local ethical committee of the University Hospital, Zurich.

### Capillary microscopy

The morphology of nailfold capillaries has been studied by intravital capillaroscopy (Leica, Glattbrugg, Switzerland) at a magnification of 50× [10]. The room temperature was maintained between 22°C and 24°C. Patients were examined in a sitting position after a resting time of at least 20 minutes. The capillaries were evaluated in accordance with the criteria of the German Association of Angiology [11]. The following changes were analysed: density of capillaries (normal 7–16 capillaries per millimetre), microhaemorrhages, dilatation of capillaries, giant capillaries, and 'irregular formations' (that is, instances where capillaries were arranged in clusters with gaps in between). A dilatation was considered to be present when the arteriolar limb of the capillary loop was thicker than 50 µm and the venous limb was thicker than 20 µm, and giant capillaries were defined as those having an apex diameter of over 50 µm [11].

### Laser Doppler fluxmetry

Skin blood flow was measured in supine subjects at the lateral epicondyle (typical FM tender point [8]), in fingertips II and III (that is, of the forefinger and middle finger) and in the lower arm (control point) using the laser Doppler technique (PeriFlux PF3; Perimed, Järfälla, Sweden), as described elsewhere [12]. A blood-pressure cuff was positioned on the upper arm and standard laser Doppler probes for skin blood flow measurements were attached to the epicondyle, fingertips II and III, and the lower arm. The resting flow was recorded 5 minutes before the cuff pressure was inflated to a suprasystolic level for 3 minutes. After release of the cuff pressure, reactive hyperaemia was recorded at the four defined areas. The time to peak flow and type of peak were evaluated in each group [13]. Peak flow corresponds to the highest flow value after release of the cuff.

**Figure 1:**
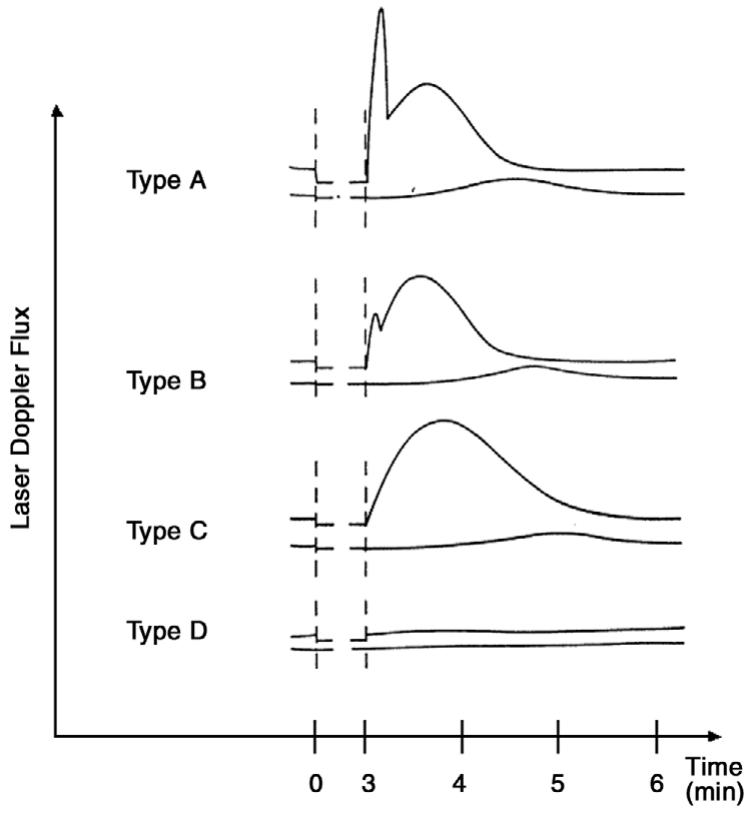
Curves depicting the four types of reactive hyperaemia measured by laser Doppler fluxmetry after occlusion of blood flow with a blood-pressure cuff for 3 minutes (occlusion marked by vertical dashed lines). (Reproduced from reference [13] with permission.)

Four types of reactive hyperaemia were identified [13] (Fig. 1). In type A, the first peak is within 23 seconds after cuff release and is followed by a second, smaller, wave. In type B, the amplitude of the second wave is greater than that of the first; the first peak is characterised by a fast dilatation of the myogen-activated arterioles and small arteries with a concomitant increase of the vessel tonus. Both types A and B are biphasic and are classified as 'normal', because so far they have been predominantly found in healthy subjects [13]. They also show the same characteristics in therapy and there is so far no proof that one type predisposes to a certain illness. Type C is monophasic; the fast initial component of the muscular reaction is absent. In type D, postocclusive reactive hyperaemia is missing. Types C and D are pathological reactions.

The Mann–Whitney *U *test was used for statistical comparison of the groups. *P *values < 0.05 were considered to be statistically significant. Means ± standard deviations are given.

## Results

### Capillary microscopy

**Figure 2:**
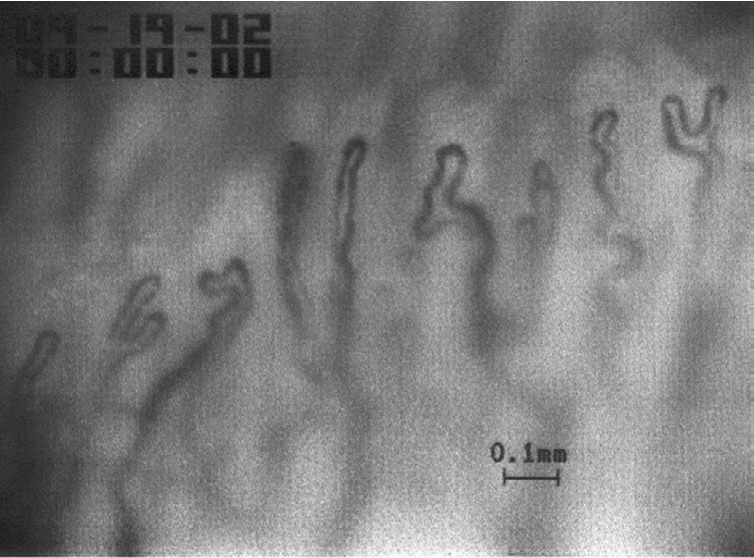
Example of capillary microscopy of the nail fold of a 44-year-old FM patient. The number of capillaries per millimetre (6) is reduced and capillary dilatations and irregular formations of the capillaries are present.

The density of capillaries per millimetre in FM patients (9.92 ± 0.19) was significantly lower than in controls (11.31 ± 0.34) (*P *< 0.001) but still within the normal range. Four or more capillary dilatations were detected in 2 of the 10 FM patients (Fig. 2), and one to three dilatations per nail fold were detected in 6 of the10 FM patients. No microhaemorrhages, giant capillaries, or avascular fields were detected in FM patients.

**Figure 3:**
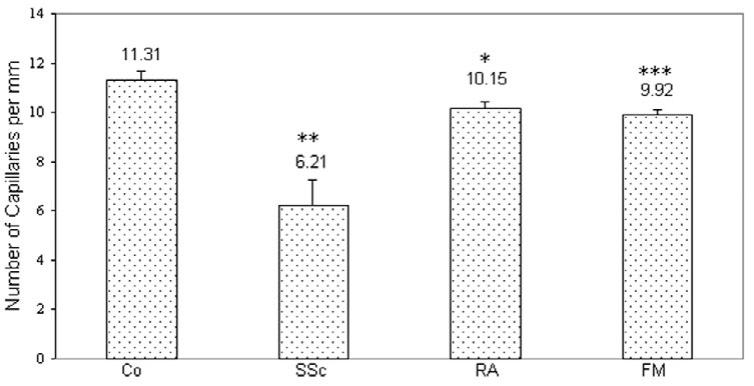
Number of capillaries (mean ± standard deviation) per millimetre in the nail fold as seen on capillary microscopy. *P < 0.05 in comparison with healthy controls (Co); **P < 0.01 in comparison with Co and with primary fibromyalgia (FM) or rheumatoid arthritis (RA) patients; ***P < 0.001 in comparison with Co.

The number of capillaries in patients with SSc (6.21 ± 1.03) was significantly lower than in healthy controls (Fig. 3) and significantly more microhaemorrhages were found in SSc patients (8 of 10) than in controls or in FM or RA patients (P < 0.01). Giant capillaries were detected only in SSc patients.

### Laser Doppler fluxmetry

**Figure 4:**
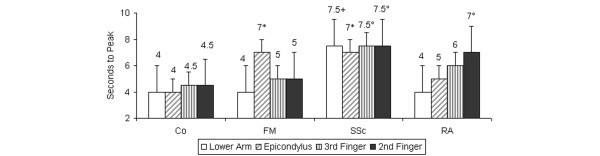
Time (s) (mean ± standard deviation) to peak capillary flow after occlusion of blood flow with a blood-pressure cuff on the upper arm for 3 minutes. Measurements were made using laser Doppler fluxmetry. In primary fibromyalgia (FM) and systemic scleroderma (SSc) patients, the time to peak in the lateral epicondyle was longer than in healthy controls (Co). In SSc patients, the times to peak in the second and third fingertips were longer than those in FM patients and healthy controls. *P < 0.001 in comparison with Co; °*P *< 0.05 in comparison with Co and with FM patients; ^+^*P *< 0.05 in comparison with Co and with FM or rheumatoid arthritis (RA) patients.

The time to peak blood flow at the lateral epicondyle was significantly longer in FM (7 ± 0.5 s) and SSc (7 ± 0.91 s) patients than in healthy controls (4 ± 0.34 s) (Fig. 4). In SSc patients, the time to the peak in the second finger (7.5 ± 1.22 s) was significantly longer than in FM patients (5 ± 0.27 s) and healthy controls (4.5 ± 0.58 s) and also in the third finger (7.5 ± 0.67 s, 5 ± 0.3 s, and 4.5 ± 0.17 s, respectively) (Fig. 4). In RA patients, the time to the peak in the second finger (7 ± 1.15 s) was significantly longer than in FM patients (5 ± 0.27 s) and healthy controls (4.5 ± 0.58 s). In the lateral epicondyle, both FM and SSc patients had longer times to peak than the healthy controls (Fig. 4).

**Figure 5:**
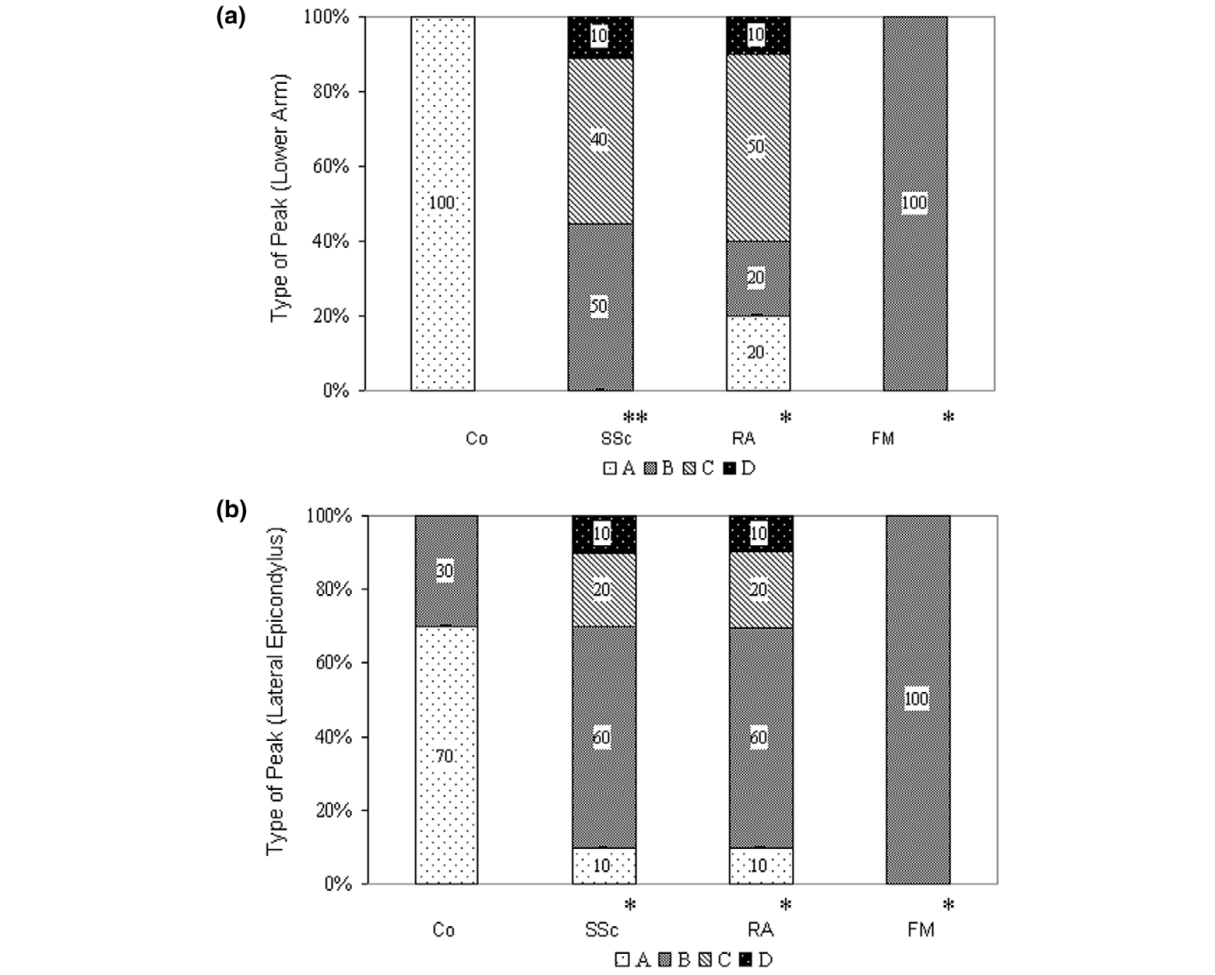
Histograms showing types of peak flow as measured using laser Doppler fluxmetry [13]. **(a) **In the lower arm and **(b) **at the lateral epicondyle [13] of patients with systemic scleroderma (SSc), rheumatoid arthritis (RA), or primary fibromyalgia (FM) and in healthy controls (Co). *P < 0.05, **P < 0.01, in comparison with Co.

All of the FM patients showed a type-B hyperaemic response in the lower-arm and epicondyle measurements (Fig. 5). The monophasic, type-C response was seen at the lateral epicondyle in 6 of 10 RA patients and 6 of 10 SSc patients. One patient with SSc and one with RA showed no postocclusive reaction (type D) either in the lower arm or at the lateral epicondyle.

**Figure 6:**
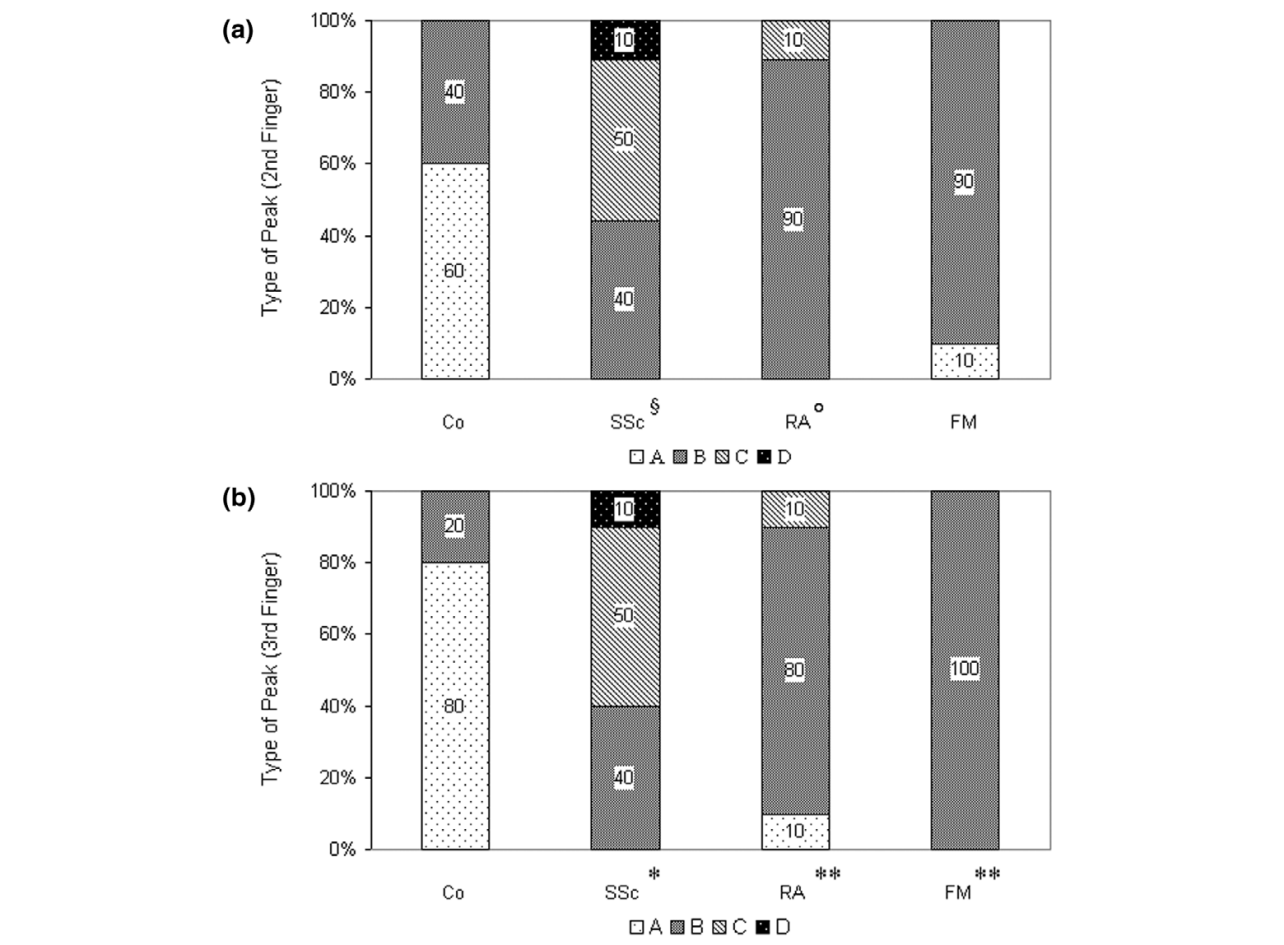
Histograms showing types of peak flow as measured using laser Doppler fluxmetry [13]. In the **(a) **second and **(b) **third fingertips of patients with systemic scleroderma (SSc), rheumatoid arthritis (RA), or primary fibromyalgia (FM) and in healthy controls (Co). °*P *< 0.05 in comparison with Co; §*P *< 0.05 in comparison with Co and FM; *P < 0.05 in comparison with Co, FM and RA; **P < 0.01 in comparison with Co.

In measurements made in the fingers, FM patients showed the postocclusive, type-B response in fingers II and III (Fig. 6a,6b), except for a type-A response in finger II in one patient. This was significantly different from the response in healthy controls (*P *< 0.01). The monophasic, type-C response was found in some patients with SSc and RA. In one patient with SSc, a type-D response was observed in all four fingers.

## Discussion

Patients with FM and SSc present functional as well as morphological changes in microcirculation, but the diseases are distinguishable by the severity of morphological pathologies of capillaries in SSc. Specific capillary abnormalities are present in patients with SSc and have a high predictive value [5]. These changes and the irregular formations in SSc may be due to microinfarcts [14]. Our results show that the density of capillaries in patients with FM is still normal but lower than in healthy controls. In an earlier study, however, it was reported that the number of capillaries is decreased [15]. Morphological abnormalities and vascular malfunction (spasms) therefore have to be discussed as possible reasons for this decreased number of capillaries. In our study, the main finding is a longer time to peak flow (reactive hyperaemia) after occlusion in FM patients than in the healthy controls. An earlier study [13] showed that 80% of healthy persons have a biphasic type of reactive hyperaemia, such as type A or B (see Fig. 1). Apart from one type-A response in the second fingertip, all of the patients with FM whom we studied were recorded as having a type-B response. This type of response is classified as 'normal', but it lacks the first, fast, myogen-activated peak. The explanation may be that FM patients have a reduced primary muscular vessel reaction whereas – and this is important – the second wave is normal.

The small number of patients in this preliminary study may be a limitation in that we may have coincidentally recorded 10 FM patients with a type-B response to occlusion of the blood flow. Further studies with more patients are needed to confirm this finding.

The missing fast component of reactive hyperaemia in our FM population is presumably due to a higher sympathetic tonus, resulting in increased vasoconstriction; this increased vasoconstriction would explain both the significantly increased time to peak, especially at the lateral epicondyle – which is a tender point in FM – and the reduced density of vessels. Earlier workers [4] advanced the idea that psychological and physical situations of stress might have an impact on this system. Local ischaemia, which may result from these proposed mechanisms in more advanced stages of the disease, could be a possible explanation for Raynaud's phenomenon in a fraction of FM patients. This local ischaemia results in an influence on spinal and supraspinal structures with sympathetic and motor efferences.

SSc patients showed also a prolonged time to peak flow at all points studied. It is well known that endothelial changes are present in SSc [14].

Both morphological and symptomatic disturbances in microcirculation can occur in FM but occur most often in SSc [16]. Functional changes have already been observed in FM, SSc, and RA [7,17]. However, the changes in FM support the hypothesis of increased sympathetic activity, and hence a functional hyperexcitability of the sympathetic nervous system [4].

## Conclusion

We have shown that functional changes of the microcirculation are present in patients with FM and this finding may be important for new treatment options in FM. A possible therapeutic strategy could be selective suppression of the sympathetic tone or the undertaking of symptomatic measures to activate the microcirculation, such as active and passive physical methods.

Because the unblinded design was a weakness of this preliminary study, blinded studies with more patients are needed to confirm our findings.

## Abbreviations

FM = primary fibromyalgia; RA = rheumatoid arthritis; SSc = systemic scleroderma.

## Competing interests

The author(s) declare that they have no competing interests.

## Authors' contributions

SM recorded measurements for all subjects and performed the capillary microscopy and laser fluxmetry. AF helped with the capillary microscopy. BA-V helped with the laser fluxmetry. UKF and RK discussed the methods and the results of the measurements. DU gave advice with respect to the study design and manuscript. HS developed the study and supervised the work of SM. All authors read and approved the final manuscript.

## Figures and Tables

**Table 1:** Characteristics of patients and controls studied.

Group, and subject no.	Risk factor	Raynaud's phenomenon	Medsger score^a^
*Patients with fibromyalgia*			
1	Obesity	-	
2	-	-	
3	-	-	
4	-	X	
5	-	X	
6	Obesity	-	
7	-	-	
8	-	-	
9	-	-	
10	-	-	
*Controls with systemic scleroderma (SSc)*			
1	-	X	4
2	-	X	3
3	-	X	2
4	-	-	1
5	-	X	2
6	Obesity	X	2
7	Ex-smoker	X	1
8	Ex-smoker	X	3
9	-	X	1
10	-	X	3
*Controls with rheumatoid arthritis*			
1	Obesity	-	
2	Hypertension	-	
3	-	-	
4	-	-	
5	-	-	
6	Obesity	-	
7	Hypertension	-	
8	-	-	
9	-	-	
10	-	-	
*Healthy controls*			
1	Obesity	-	
2	Ex-smoker	-	
3	-	-	
4	-	-	
5	-	-	
6	Ex-smoker	-	
7	Obesity	-	
8	-	-	
9	-	-	
10	-	-	

^a^Assigned to SSc patients only; indicates severity of the disease, from 0 to 4 (none to end stage) [9]. -, Not present; X, present.
